# High-risk human papillomavirus-associated vulvar neoplasia among women living with human immunodeficiency virus in Zambia

**DOI:** 10.4102/ajlm.v11i1.1563

**Published:** 2022-05-12

**Authors:** Fred Maate, Peter Julius, Stepfanie Siyumbwa, Leeya Pinder, Trevor Kaile, Mulindi Mwanahamuntu, Groesbeck Parham

**Affiliations:** 1 Department of Pathology and Microbiology, Adult and Emergency Hospital, University Teaching Hospitals, Lusaka, Zambia; 2 Department of Pathology and Microbiology, School of Medicine, University of Zambia, Lusaka, Zambia; 3 Department of Obstetrics and Gynecology, University of Washington, Seattle, Washington, United States; 4 Department of Obstetrics and Gynaecology, Women and Newborn Hospital, University Teaching Hospitals, Lusaka, Zambia; 5 Department of Obstetrics and Gynecology, University of North Carolina at Chapel Hill, Chapel Hill, North Carolina, United States

**Keywords:** vulvar neoplasia, vulvar cancer, human papillomavirus virus, HIV, Zambia

## Abstract

**Background:**

Globally, women living with HIV have a higher risk of vulvar neoplasia than HIV-negative women. Vulvar neoplasia among women living with HIV has not previously been characterised in Zambia.

**Objective:**

This study determined the clinical and pathologic features of vulvar neoplasia among women living with HIV at the University Teaching Hospital, Lusaka, Zambia.

**Methods:**

We conducted a cross-sectional study of vulvar lesions among 53 women living with HIV who presented with vulvar lesions between July 2017 and February 2018. The study assessed clinical and histological characteristics and prevalence of high-risk human papillomavirus (HRHPV).

**Results:**

Twenty-one patients were diagnosed with vulvar squamous cell carcinoma (VSCC), 20 with usual vulvar intraepithelial neoplasm (uVIN), and the rest with either benign lesions or non-neoplastic lesions (NNL). Participants’ mean age was 40 years. Patients with VSCC were significantly older than those with NNL (mean (s.d.): 43 (21) vs 33 (10), *p* = 0.004). The prevalence of HRHPV was 88.9% in VSCC patients and 100.0% in high-grade squamous intraepithelial lesion patients. HPV16 was the most common (52.6%) genotype. The clinical features of neoplasia were similar to those of NNL.

**Conclusion:**

VSCC was significantly more common among women aged ≥ 40 years. HRHPV in VSCC and high-grade squamous intraepithelial lesions was high. Women with vulvar lesions, especially those aged > 40 years, should be evaluated for vulvar cancer. Young girls should be vaccinated to prevent vulvar cancer.

## Introduction

Vulvar intraepithelial neoplasia (VIN) is a precursor of vulvar squamous cell carcinoma (VSCC).^1^ Two types of VIN have been described: differentiated VIN and usual vulvar intraepithelial neoplasia (uVIN). Usual VIN occurs in young women and is associated with high-risk human papillomavirus (HRHPV) infection.^2,3^ Differentiated VIN occurs in older women and is not associated with HRHPV^4^ but is associated with chronic inflammatory disorders such as lichen sclerosus and squamous cell hyperplasia.^5,6^

By definition, HRHPVs are those that are associated with cervical and anogenital cancers, including vulvar cancer.^7^ In 2005, the International Agency for Research on Cancer defined 13 genotypes of HPV as high-risk/carcinogenic; these include genotypes 16, 18, 31, 33, 35, 39, 45, 51, 52, 56, 58, 59 and 68.^8^ Other genotypes not related to cancer, such as HPV6 and 11, are said to be low-risk, and these are known to cause benign exophytic lesions such as condyloma acuminatum.^9^

VSCC is the most common histologic type of vulvar cancer. It accounts for 95% of malignant tumours of the vulva.^2,10^ Two forms of VSCC have been established, including the HRHPV-associated warty and basaloid VSCC and the non-HRHPV-associated keratinising VSCC.^11,12^ Warty and basaloid VSCC arise from uVIN, while keratinising VSCC arises from differentiated VIN.^5^

Globally, vulvar cancer is more common among elderly women above 60 years.^13,14,15^ However, over the last two decades, its incidence has increased, particularly among young women, and this is attributed to infection with HPV.^16,17,18,19^

In Zambia, vulvar cancer was the second most common gynaecologic malignancy in 2018.^20^ Between 2007 and 2014, the age-standardised incidence rate of the disease was 1.5 per 100 000 women.^21^ Also, it was reported recently that the disease peaked in the reproductive age group (20–40 years old).^21^ There is substantial evidence that HIV positivity is associated with HPV infection; one in every four women with vulvar cancer in Zambia was HIV-positive.^21,22,23^ However, little is known about the clinical and pathological presentation of vulvar cancer and other vulvar lesions in Zambia, particularly among women living with HIV.

This study aimed to compare the clinicopathologic features and risk factors of vulvar neoplasia with those of non-neoplastic lesions (NNL) among women living with HIV. It also aimed to determine the prevalence of HRHPV infection among vulvar neoplasia patients. This information would provide etiological and clinical context to vulvar neoplasia in Zambia and identify areas for further research and any challenges associated with vulvar neoplasia.

## Methods

### Ethical considerations

This study was approved by the University of Zambia Biomedical Research Ethics Committee (reference: 015-12-16), the National Health Research Authority, and the University of North Carolina at Chapel Hill Institutional Review Board (reference: IRB: 16-3422). Permission to conduct the study was obtained from the management of the University Teaching Hospitals. Participants provided written informed consent to participate in the study. To maintain confidentiality, no identifying information was collected and participant data were de-identified using unique codes before storage in a password-protected computer accessed only by the principal investigator. Data from participants who withdrew from the study were completely removed.

### Study design and setting

This was a cross-sectional hospital-based clinical and laboratory study conducted between July 2017 and February 2018 at the University Teaching Hospital’s Women and New Born Hospital, Lusaka, Zambia. The hospital is the country’s largest public tertiary hospital for obstetric, gynaecologic, and neonatal care.

Women attending the University Teaching Hospital’s gynaecologic clinic between July 2017 and February 2018 were recruited. The sample size was determined using the Fisher formula: Z^2^ *p* (1 − *p*)/d^2^ based on an assumed prevalence (*p*) of 3.6%, a Z score of 1.96 and a margin of error (d) of ±5%. Women who were aged 18 years or older, presented with a vulvar lesion and were documented as HIV-positive were included in the study. Women who were unwilling to undergo a punch biopsy and had a previous diagnosis of vulvar cancer and a history of bleeding disorder were excluded.

A total of 56 women were approached, of which 53 were recruited for the study; two were ineligible and one did not consent.

Demographic information, frequency of selected risk factors (smoking, age at first sexual intercourse, number of lifetime sexual partners, alcohol use, and oral contraceptive use), and a comprehensive clinical history were obtained using a structured questionnaire. CD4 count tests were conducted or results obtained from patients’ medical records if the test was done within the last six months. Clinical examinations of the vulva were conducted and the characteristics of the vulvar lesions were recorded. The sizes of the vulvar lesions were determined using a measuring tape.

### Biopsy and histopathological analysis

Tissues for histologic diagnosis were collected using Keyes’ punch biopsy forceps under local anaesthesia and aseptic conditions. The tissues were preserved in 10% neutral buffered formalin and transported to the pathology laboratory of the University Teaching Hospital’s Adult and Emergency Hospital. The tissues were processed in the laboratory according to standard protocols, after which the histologic evaluation was conducted by three pathologists. Based on the classification of the International Society for the Study of Vulvovaginal Disease (2015),^24^ the lesions were classified as NNL, condylomata acuminatum, uVIN, or VSCC. NNL types included acute non-specific inflammation, chronic non-specific inflammation and herpes simplex ulcer. Usual VIN types included low-grade squamous intraepithelial lesion (LSIL) and high-grade squamous intraepithelial lesion (HSIL). The pathologists diagnosed the vulvar lesions as LSIL if the dysplastic cellular changes were limited to the lower third of the epithelium, and HSIL if the dysplastic cellular changes involved up to or greater than the upper two-thirds of the epithelium.

### Detection of high-risk human papillomavirus

Punch biopsies for the polymerase chain reaction test were collected during the collection of tissue for histology. Each tissue was preserved in a 1.5 mL microcentrifuge tube containing Qiagen Buffer ATL (Qiagen, Germantown, Maryland, United States) and stored in a refrigerator at –80 °C. Samples were allowed to defrost at room temperature, mixed with 20 µL of proteinase K, and then incubated and vortexed to ensure a complete mixture. Total DNA was extracted from the pre-lysed samples using the Nuclisens^®^ EasyMag™ automated extraction machine (BioMerieux, Inc, Marcy I’Etoile, France) according to the manufacturer’s protocol. The elution volume was 100 µL. Polymerase chain reaction for the detection of high-risk HPV was carried out using the Xpert^®^ HPV platform (Cepheid, Sunnyvale, California, United States). To do this, 100 µL of each extracted sample was mixed with 900 µL of the Thin Prep solution (Hologic, Marlborough, Massachusetts, United States), allowed to homogenise, and then transferred into a cartridge. The cartridge was loaded into the Gene Xpert PCR machine (Cepheid, Sunnyvale, California, United States) and run according to the manufacturer’s protocol. Results were exported to a disc and transferred to a computer. Xpert HPV detects the presence of high-risk HPV and the following HPV genotypes: HPV16, HPV18_45, and other HPVs, including P3 (HPV31, HPV33, HPV35, HPV52, HPV58), P4 (HPV51, HPV59), and P5 (HPV39, HPV56, HPV66, HPV68).

### Statistical analysis

All data management was done using Excel (Microsoft Corporation, Redmond, Washington, United States) and data analyses were carried out using Statistical Package for Social Sciences version 22 (International Business Machines Corporation, Armonk New York, United States). Clinical features (symptoms, and signs), demographic features and risk factors of uVIN and VSCC were compared with those of NNL using Chi-square or Fisher’s exact tests of association. We excluded condyloma acuminatum from the comparative analysis because of its benign nature. The continuous variables (age, age at first sexual intercourse, duration of antiretroviral therapy [ART], number of lifetime sexual partners, and duration of symptoms in years) were tested for normality using the Shapiro-Wilk test. The means of the continuous variables that were normally distributed (age [*p* = 0.969] and duration of ART [*p* = 0.070]) were compared using Levine’s *T*-test between the diagnostic categories VSCC and NNL, as well as between uVIN and NNL. Medians and interquartile ranges were reported for variables (number of lifetime sexual partners and duration of symptoms) that were not normally distributed. The median duration of symptoms and the median number of lifetime sexual partners were compared using Mood’s median test between the diagnostic categories VSCC and NNL, as well as between uVIN and NNL.

## Results

### Histologic diagnoses

**FIGURE 1:**
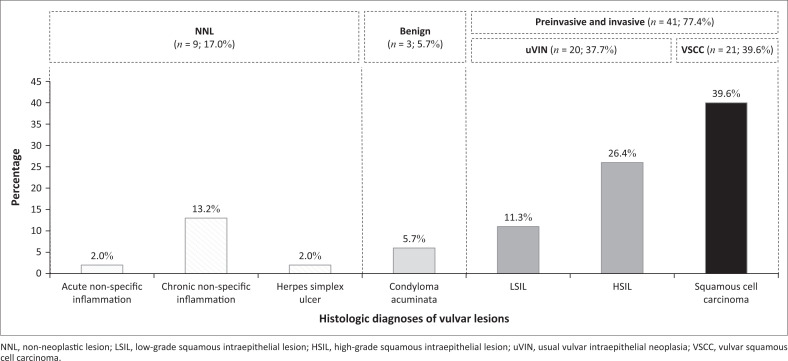
Histological diagnosis of vulvar lesions from women attending the University Teaching Hospital’s gynaecologic clinic, Zambia, 2018.

**FIGURE 2:**
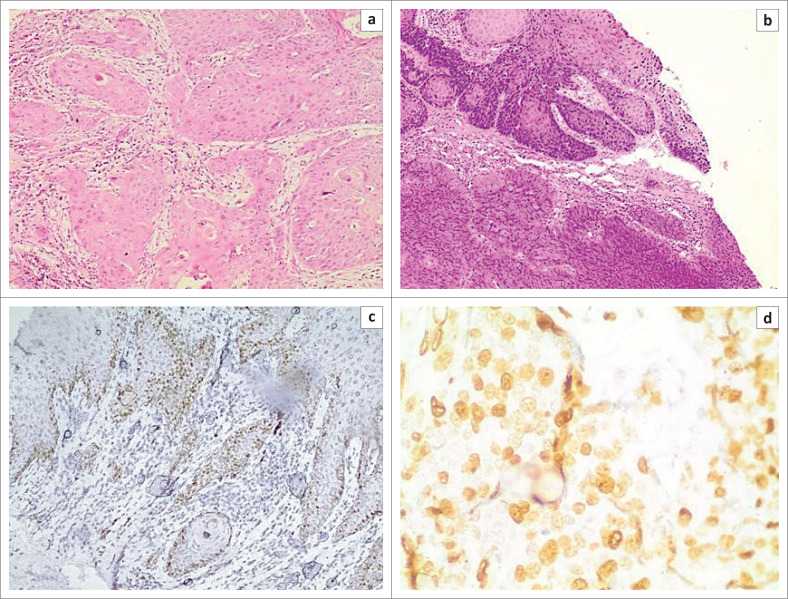
P53 and Ki-67 immunostaining photomicrographs of warty vulvar squamous cell carcinoma and basaloid vulvar squamous cell carcinoma among women attending the University Teaching Hospital, Zambia, 2018. (a) Warty vulvar squamous cell carcinoma: Nests of malignant squamous cells are noted surrounded by a desmoplastic stroma. (b) Basaloid vulvar squamous cell carcinoma: Sheets of cells with hyperchromatic nuclei and scant to no cytoplasm are seen with overlying atypical surface epithelium. (c) P53 immunostaining in warty vulvar squamous cell carcinoma. Focal staining is noted. (d) Ki-67 stain. A diffuse nuclear staining pattern is noted.

Histologic types of vulvar lesions included VSCC (21/53, 39.6%), uVIN (20/53, 37.7%), NNL (9/53, 17.0%) and condyloma acuminatum (3/53, 5.7%). VSCC followed by uVIN were the sole types of cancer and premalignant disease identified (Figure 1). Two types of uVIN were reported: HSIL (14/20, 70.0%) and LSIL (6/20, 30.0%). The types of NNL included chronic non-specific inflammation (7/9, 77.8%), acute inflammation (1/9, 11.1%), and herpes simplex ulcer (1/9, 11.1%) (Figure 2). Data from the three participants with condyloma acuminatum were excluded from downstream analyses. The VSCC cases were either warty (19/21, 90.5%) or basaloid types (2/21, 9.5%). No keratinising squamous cell carcinoma was observed. Among the warty VSCC, two cases (2/19, 10.5%) were moderately differentiated (grade 2) and 17 (17/19, 89.5%) were poorly differentiated (grade 3). On the other hand, all of the basaloid (2/2, 100.0%) VSCCs were poorly differentiated (grade 3). Among the VSCC cases, 16 (76.2%) had concurrent HSIL, 19 (90.5%) had a high Ki-67 index (30% or more), one (4.8%) was positive for p53, one (4.8%) had vascular invasion, and one (4.8%) had concurrent herpes simplex viral cytopathic effects.

### Demographic characteristics of participants with VSCC and uVIN compared to those with NNL

Approximately three-quarters (39/50, 78.0%) of the study participants were residents of Lusaka; 21/50 (42.0%) were married and 32/50 (64.0%) were employed (Table 1). There was no difference in employment (uVIN vs NNL: *p* = 0.205; VSCC vs NNL: *p* = 0.443) or marital status (uVIN vs NNL: *p* = 0.439; VSCC vs NNL: *p* = 0.389) between the study participants with NNL and participants with uVIN or VSCC. Over half (27/50, 54.0%) were aged between 20 years and 39 years, and 46.0% (23/50) were 40 years or older. The mean age of the participants was 40 years (range: 23–63 years). VSCC was significantly more common among participants aged ≥ 40 years (*p* = 0.014) than among participants aged between 20 and 39 years. The mean age was also significantly higher among participants with VSCC (43 vs 33 years, *p* = 0.004) compared to those with NNL. There was no difference between the mean ages of participants with uVIN and NNL (uVIN: 40 years vs NNL: 33 years, *p* = 0.110). The majority (23/50, 46.0%) of participants reported primary level as their highest level of education; however, the tests of association could not be performed as the assumptions for χ^2^ and Fisher’s exact tests were not met.

**TABLE 1:** Characteristics of participants diagnosed with usual uVIN, VSCC and NNL at the University Teaching Hospital’s gynaecologic clinic, Zambia, 2018.

Characteristics of participants	All (*N* = 50)					NNL (*N* = 9)					uVIN (*N* = 20)					*p* (NNL vs uVIN)	VSCC (*N* = 21)					*p* (NNL vs VSCC)
	*n*	%	Mean ± s.d.	Median	IQR	*n*	%	Mean ± s.d.	Median	IQR	*n*	%	Mean ± s.d.	Median	IQR		*n*	%	Mean ± s.d.	Median	IQR	
**Age**																						
20–39	27	54.0	-	-	-	8	88.9	-	-	-	12	60.0	-	-	-	0.201*	7	33.3	-	-	-	0.014*
40 and above	23	46.0	-	-	-	1	11.1	-	-	-	8	40.0	-	-	-	-	14	66.7	-	-	-	-
Age†	-	-	40 ± 10	-	-	-	-	33 ± 10	-	-	-	-	40 ± 20	-	-	0.110	-	-	43 ± 21	-	-	0.004
**Residence**																						
Non-Lusaka	11	22.0	-	-	-	1	11.1	-	-	-	6	30.0	-	-	-	0.382*	4	19.0	-	-	-	1.000*
Lusaka	39	78.0	-	-	-	8	88.9	-	-	-	14	70.0	-	-	-	-	17	81.0	-	-	-	-
**Marital status**																						
Single	7	14.0	-	-	-	3	33.3	-	-	-	2	10.0	-	-	-	0.439	2	9.5	-	-	-	0.389
Married	21	42.0	-	-	-	3	33.3	-	-	-	10	50.0	-	-	-	-	8	38.1	-	-	-	-
Divorced/separated	13	26.0	-	-	-	1	11.1	-	-	-	5	25.0	-	-	-	-	7	33.3	-	-	-	-
Widowed	9	18.0	-	-	-	2	22.1	-	-	-	3	15.0	-	-	-	-	4	19.0	-	-	-	-
**Employment**																						
Unemployed	18	36.0	-	-	-	5	55.6	-	-	-	5	25.0	-	-	-	0.205*	8	38.1	-	-	-	0.443*
Employed	32	64.0	-	-	-	4	44.4	-	-	-	15	75.0	-	-	-	-	13	61.9	-	-	-	-
**Education**																						
None	4	8.0	-	-	-	1	11.1	-	-	-	2	10.0	-	-	-	N/a	1	4.8	-	-	-	N/a
Primary	23	46.0	-	-	-	2	22.2	-	-	-	7	35.0	-	-	-	-	14	66.7	-	-	-	-
Secondary	18	36.0	-	-	-	5	55.6	-	-	-	8	40.0	-	-	-	-	5	23.8	-	-	-	-
Tertiary	5	10.0	-	-	-	1	11.1	-	-	-	3	15.0	-	-	-	-	1	4.8	-	-	-	-
**ART ever**																						
Yes	48	96.0	-	-	-	7	77.8	-	-	-	20	100.0	-	-	-	N/a	21	100.0	-	-	-	N/a
**ART duration**																						
< 1 year	9	18.0	-	-	-	2	28.6	-	-	-	5	25.0	-	-	-	1.000	2	9.5	-	-	-	0.253
> 1 year	39	78.0	-	-	-	5	71.4	-	-	-	15	75.0	-	-	-	-	19	90.5	-	-	-	-
Years	-	-	7 ± 4	-	-	-	-	5 ± 4	-	-	-	-	7 ± 5	-	-	-	-	-	7 ± 3	-	-	-
CD4 count (cells/µL)†	-	-	387.2 ± 237.2	-	-	-	-	381.6 ± 294.1	-	-	-	-	359.5 ± 225.7	-	-	0.835	-	-	414.0 ± 233.1	-	-	0.757
**Duration of symptoms**																						
Less than a year	13	26.0	-	-	-	5	55.6	-	-	-	4	20.0	-	-	-	0.088*	4	19.0	-	-	-	0.082*
More than a year	37	74.0	-	-	-	4	44.4	-	-	-	16	80.0	-	-	-	-	17	81.0	-	-	-	-
Duration of symptoms (years)‡	-	-	-	3	1–6	-	-	-	2	1–9	-	-	4	1.5–5.5	-	0.582	-	-	-	2	1–6	1.000
History of smoking cigarettes	3	6.0	-	-	-	1	11.1	-	-	-	1	5.0	-	-	-	0.532^#^	1	4.8	-	-	-	0.517**
History of taking alcohol	16	32.0	-	-	-	2	22.2	-	-	-	7	35.0	-	-	-	0.675*	7	33.3	-	-	-	0.681**
History of using oral contraceptives	41	82.0	-	-	-	7	77.8	-	-	-	18	90.0	-	-	-	0.568*	16	76.2	-	-	-	1.000*
**Age range at first sexual intercourse**																						
Below 15	12	24.0	-	-	-	1	11.1	-	-	-	6	30.0	-	-	-	N/a	5	23.8	-	-	-	N/a
16–20	30	60.0	-	-	-	6	66.7	-	-	-	13	65.0	-	-	-	-	11	52.4	-	-	-	-
21–25	7	14.0	-	-	-	2	22.2	-	-	-	1	5.0	-	-	-	-	4	19.0	-	-	-	-
Unknown	1	2.0	-	-	-	0	0.0	-	-	-	0	0.0	-	-	-	-	1	4.8	-	-	-	-
Median age at first sexual intercourse†	-	-	-	18	18–20	-	-	-	19	18–21	-	-	-	18	15–18	0.048	-	-	-	17	16–19	0.226
Number of lifetime sexual partners‡	-	-	-	4	2–6	-	-	-	3	2–4	-	-	-	4	2–6	0.454	-	-	-	4	2–6	0.419
**History of cervical cancer screening**																						
Yes	33	66.0	-	-	-	7	77.8	-	-	-	13	65.0	-	-	-	0.694	13	61.9	-	-	-	1.000
**Previously diagnosed with cervical cancer**																						
Yes	2	4.0	-	-	-	0	0.0	-	-	-	1	5.0	-	-	-	N/a	1	5.0	-	-	-	N/a
**Symptoms experienced**																						
Itching	44	88.0	-	-	-	9	100.0	-	-	-	18	90.0	-	-	-	N/a	17	81.0	-	-	-	N/a
Pain	39	78.0	-	-	-	6	66.7	-	-	-	13	65.0	-	-	-	1.000*	20	95.2	-	-	-	0.069*
Painful sex	5	10.0	-	-	-	0	0.0	-	-	-	2	10.0	-	-	-	N/a	3	14.3	-	-	-	N/a
Bleeding	18	36.0	-	-	-	2	22.2	-	-	-	3	15.0	-	-	-	0.633*	13	61.9	-	-	-	0.109*
Lesion discharge	33	66.0	-	-	-	6	67.7	-	-	-	9	45.0	-	-	-	0.427*	18	85.7	-	-	-	0.329*
**First symptom experienced**																						
Itching	23	46.0	-	-	-	4	44.4	-	-	-	9	45.0	-	-	-	1.000	10	47.6	-	-	-	1.000
Pain	3	6.0	-	-	-	0	0.0	-	-	-	1	5.0	-	-	-	N/a	2	9.5	-	-	-	N/a
Painful sex	1	2.0	-	-	-	1	11.1	-	-	-	0	0.0	-	-	-	N/a	0	0.0	-	-	-	N/a
Ulcer	4	8.0	-	-	-	2	22.2	-	-	-	0	0.0	-	-	-	0.089*	2	9.5	-	-	-	0.563*
Black patches	1	2.0	-	-	-	0	0.0	-	-	-	1	5.0	-	-	-	N/a	0	0.0	-	-	-	N/a
Growth	18	36.0	-	-	-	2	22.2	-	-	-	9	45.0	-	-	-	0.412*	7	33.3	-	-	-	0.681*
**Sites of vulvar lesions**																						
Labia majora	42	84.0	-	-	-	9	100.0	-	-	-	14	70.0	-	-	-	N/a	19	90.5	-	-	-	N/a
Labia minora	36	72.0	-	-	-	6	66.7	-	-	-	14	70.0	-	-	-	1.000*	16	76.2	-	-	-	0.666*
Clitoris	14	28.0	-	-	-	3	33.3	-	-	-	4	20.0	-	-	-	0.642*	7	33.3	-	-	-	1.000*
**Involvement of other sites**																						
Vagina	3	6.0	-	-	-	0	0.0	-	-	-	0	0.0	-	-	-	N/a	3	14.3	-	-	-	N/a
Perianal skin	4	8.0	-	-	-	0	0.0	-	-	-	2	10.0	-	-	-	-	2	9.5	-	-	-	-
Anus	1	2.0	-	-	-	0	0.0	-	-	-	0	0.0	-	-	-	-	1	16.7	-	-	-	-
Gluteal skin	1	2.0	-	-	-	1	11.1	-	-	-	0	0.0	-	-	-	-	0	0.0	-	-	-	-
**Number of foci**																						
Unifocal	9	18.0	-	-	-	0	0.0	-	-	-	4	20.0	-	-	-	N/a	5	23.8	-	-	-	N/a
Multifocal	41	82.0	-	-	-	9	100.0	-	-	-	16	80.0	-	-	-	-	16	76.2	-	-	-	-
**Size of vulvar lesions (of the largest, if multiple)**																						
≤ 2 cm	8	16.0	-	-	-	1	11.1	-	-	-	5	25.0	-	-	-	0.633*	2	9.5	-	-	-	0.672**
> 2 cm	42	84.0	-	-	-	8	88.9	-	-	-	15	75.0	-	-	-	-	19	90.5	-	-	-	-
**Type of vulvar lesion†**																						
Smooth exophytic	10	20.0	-	-	-	2	22.2	-	-	-	2	10.0	-	-	-	N/a	6	28.6	-	-	-	N/a
Warty	17	34.0	-	-	-	3	33.3	-	-	-	11	55.0	-	-	-	-	3	14.3	-	-	-	-
Fungating	12	24.0	-	-	-	1	11.1	-	-	-	2	10.0	-	-	-	-	9	42.9	-	-	-	-
Flat	3	6.0	-	-	-	0	0.0	-	-	-	3	15.0	-	-	-	-	0	0.0	-	-	-	-
Ulcerated	8	16.0	-	-	-	3	33.3	-	-	-	2	10.0	-	-	-	-	3	14.3	-	-	-	-

ART, antiretroviral therapy; N/a, chi-square assumptions not met; s.d., standard deviation; IQR, interquartile range; NNL, non-neoplastic lesion; uVIN, usual vulvar intraepithelial neoplasia; VSCC, vulvar squamous cell carcinoma.

* , Fisher’s test: Expected value assumption met and 2-sided significance reported;

** , Fisher’s test:expected count assumption not met.

† , *T*-test reported;

‡ , Moods test.

### Risk factors among participants with VSCC and uVIN compared to those with NNL

There was no association between the selected risk factors and the diagnosis of VSCC or uVIN: history of alcohol intake (uVIN vs NNL: *p* = 0.675; VSCC vs NNL: *p* = 0.681), oral contraceptive intake (uVIN vs NNL: *p* = 0.568; VSCC vs NNL: 1.000), cigarette smoking (uVIN vs NNL: *p* = 0.532; VSCC vs NNL: 0.517). The median age at first sexual intercourse was significantly lower among participants with uVIN than those with NNL (uVIN: 18 years vs NNL: 19 years; *p* = 0.048). Only a few (3/50, 6.0%) participants reported smoking. The median number of lifetime sexual partners reported among all participants was four (95% confidence interval [CI]: 2–6); four partners (95% CI: 2–6) among participants with VSCC, four partners (95% CI: 2–6) among participants with uVIN and three partners (95% CI: 2–4) among participants with NNL.

### HIV characteristics and history of cervical cancer screening

All participants diagnosed with uVIN (20/20, 100.0%) and VSCC (21/21, 100.0%) were on ART, while seven (7/9; 77.8%) of those diagnosed with NNL were on ART. The majority of the participants (39/50, 78.0%) had been on ART for longer than one year (mean: 7 ± 4 years). The CD4 count distribution was approximately normal (Shapiro-Wilk test: 0.952 [degree of freedom{*df*} = 46], *p* = 0.055). The overall mean CD4 count was 387.2 ± 237.2 cells/µL. The average CD4 count was 414.0 ± 233.1 cells/µL among participants with VSCC, 359.5 ± 225.7 cells/µL among participants with uVIN, and 381.6 ± 294.1 cells/µL among participants with NNL. There was no significant difference in mean CD4 counts between participants with NNL and those with uVIN (*t* = –0.210, *p* = 0.835) or VSCC (*t* = 0.312, *p* = 0.757).

In terms of history of cervical cancer screening, there was no difference between uVIN and NNL (13/20 (65.0%) vs 7/9 (77.8%); *p* = 0.694) or between VSCC and NNL (13/21 [61.9%] vs 13/20 [65.0%]; *p* = 1.000). Among those who were screened for cervical cancer, two of them reported that they had been diagnosed with cervical cancer (one with uVIN and the other with VSCC).

### Symptoms of participants with uVIN, VSCC and NNL

Approximately three-quarters (37/50, 74.0%) of the participants had had vulvar symptoms for at least 1 year. Although a larger proportion of the participants with VSCC (17/21, 80.9%; *p* = 0.082) and uVIN (16/20, 80.0%; *p* = 0.088) had symptoms for ≥ 1 year compared to participants with NNL (4/9, 44.4%), these differences were not statistically significant. Among all the participants, the most frequently reported first symptom was itching (23/50, 46.0%). The frequency of itching as a first symptom was similar across all groups of participants (uVIN: 9/20, 45.0%; NNL: 4/9, 44.4%; VSCC: 10/21, 47.6%). The overall most common symptoms reported among the study participants were itching (44/50, 88.0%) and pain (39/50, 78.0%). There was no significant association between the diagnostic groups and symptoms experienced.

### Location, sizes and types of lesions

Overall, most lesions involved the labia majora (42/50, 84.0%), followed by the labia minora (36/50, 72.0%) and clitoris (14/50, 28.0%). The labia majora was affected in all women with NNL (9/9, 100%), 90.0% (19/21) of women with VSCC, and 70.0% (14/20) of women with uVIN. The vagina, perineum and anus were also involved in participants with uVIN and VSCC. Overall, the majority of participants (41/50, 82.0%) had multifocal rather than unifocal lesions. All NNL were multifocal; comparatively, 18 (80.0%) women with uVIN, and 16 (76.2%) women with VSCC had multifocal lesions. The majority of the participants (42/50, 84%) had vulvar lesion sizes > 2 cm. Nineteen (19/21; 90.5%) women with VSCC had lesions > 2 cm compared to eight (88.9%) women with NNL, and 15 (75.0%) women with uVIN. The overall most common type of vulvar lesion was the warty type (17/50, 34.0%), followed by the fungating (12/50, 24.0%), smooth exophytic (10/50, 20.0%), ulcerated (8/50, 16.0%) and flat (3/50, 6.0%) types. Among women with uVIN, 11/20 (55.0%) lesions were warty, and 3/20 (15.0%) were flat; smooth exophytic, fungating, and ulcerated lesions each accounted for 2/20 (10.0%) of the cases. On the other hand, the most common type of vulvar lesion among participants with VSCC was the fungating type (9/21, 42.9%), followed by the smooth exophytic lesion (6/21, 28.6%); warty and ulcerated lesions each accounted for 14.3% (3/21).

### Prevalence of HRHPV infection among participants with neoplastic lesions

Among the samples collected for HRHPV DNA analysis, we were able to extract sufficient DNA from 38 tissue samples; these included samples from 17 patients with uVIN, 18 with VSCC and three with condylomata acuminatum. The prevalence of any type of HRHPV among all participants was 81.6% (31/38); 82.4% (14/17) among women with uVIN (1/4, 25% in LSIL, and 13/13, 100% in HSIL) and 88.9% (16/18) among women with VSCC (Table 2). The most prevalent genotype of HRHPV among all participants was HPV16 (20/38, 52.6%), followed by P3 (13/38, 34.2%) (Table 3). Only HPV16, P3 and P5 genotypes were present in HSIL; only HPV16 was detected in LSIL cases. The HPV genotypes that were detected in VSCC cases were HPV16, HPV18_45, P3 and P5. HPV16 occurred as a co-infection with other HPVs (P3 and P5) in both uVIN (HSIL) and VSCC.

**TABLE 2:** Prevalence of high-risk human papillomavirus among women with condyloma acuminatum, uVIN (LSIL, HSIL) and VSCC at the University Teaching Hospital’s gynaecologic clinic, Zambia, 2018.

Histologic diagnosis	Prevalence of HRHPV											
	Any HRHPV		HPV16		HPV18_45		Other HPVs					
							P3		P4		P5	
	*n*	%	*n*	%	*n*	%	*n*	%	*n*	%	*n*	%
All (*n* = 38)	31	81.6	20	52.6	1	2.6	13	34.2	1	2.6	4	10.5
CA (*n* = 3)	1	33.3	0	0.0	0	0.0	1	33.3	1	33.3	1	33.3
uVIN (*n* = 17)	14	82.4	11	64.7	0	0.0	5	29.4	0	0.0	2	11.8
LSIL (*n* = 4)	1	25.0	1	25.0	0	0.0	0	0.0	0	0.0	0	0.0
HSIL (*n* = 13)	13	100	10	76.9	0	0.0	5	38.5	0	0.0	2	15.4
VSCC (*n* = 18)	16	88.9	8	44.4	1	5.6	8	44.4	1	5.6	1	5.6

uVIN, usual vulvar intraepithelial neoplasia; LSIL, low-grade squamous intraepithelial lesion; HSIL, high-grade squamous intraepithelial lesion; VSCC, vulvar squamous cell carcinoma; CA, condylomata acuminatum; HRHPV, high-risk human papillomavirus; HPV, human papillomavirus.

Note: P3 includes HPV31, 33, 35, 52 and 58; P4 includes HPV51 and 59; P5 includes HPV39, 56, 66 and 68.

**TABLE 3:** Frequency of high-risk human papillomavirus genotypes among women with condylomata acuminatum, uVIN and VSCC at the University Teaching Hospital’s gynaecologic clinic, Zambia, 2018.

HPV Genotype	CA (*N* = 1)		uVIN (*N* = 14)		LSIL (*N* = 1)		HSIL (*N* = 13)		VSCC (*N* = 16)		Total (*N* = 31)	
	*n*	%	*n*	%	*n*	%	*n*	%	*n*	%	*n*	%
HPV16	0	0.0	7	50.0	1	100.0	6	46.2	6	37.5	13	41.9
HPV18_45	0	0.0	0	0.0	0	0.0	0	0.0	1	6.3	1	3.2
P3	0	0.0	3	21.4	0	0.0	3	23.1	7	43.8	10	32.3
HPV16 and others (P3)	0	0.0	2	14.3	0	0.0	2	15.4	0	0.0	2	6.5
HPV16 and others (P4)	0	0.0	0	0.0	0	0.0	0	0.0	1	6.3	1	3.2
HPV16 and others (P5)	0	0.0	2	14.3	0	0.0	2	15.4	0	0.0	2	6.5
HPV16 and others (P3 and P5)	0	0.0	0	0.0	0	0.0	0	0.0	1	6.3	1	3.2
Other HPVs (P3, P4 and P5)	1	-	0	0.0	0	0.0	0	0.0	0	0.0	1	3.2

CA, condyloma acuminatum, LSIL, low-grade squamous intraepithelial lesion, HSIL, high-grade squamous intraepithelial lesion, VSCC, vulvar squamous cell carcinoma; HRHPV, high-risk human papillomavirus; HPV, human papillomavirus.

## Discussion

To the best of our knowledge, this is the first study to describe vulvar lesions and to compare vulvar neoplasia with NNL among any cohort of women living with HIV at any hospital in Zambia. Most of the studies in other African countries largely focused on vulvar cancer or HPV.^22,25^ Among the cohort of women living with HIV in our study, both neoplastic (condylomata acuminatum, uVIN, and VSCC) and NNL were detected. This study showed that VSCC cannot be distinguished from NNL on account of clinical features, although VSCC was associated with women aged ≥ 40 years. The prevalence of HRHPV in both VSCC and uVIN (accounted for by HSIL) was high.

VSCC was significantly more common among women aged ≥ 40 years compared to NNL. Similarly, a study in Tunisia found that vulvar cancer was more common in older women (86.9% of patients were more than 55 years old) although none of the patients was HIV-positive.^26^ Clinicians should therefore evaluate and biopsy the vulva in women who present with vulvar symptoms, especially those who are older than 40 years. The ages of women affected with uVIN in our study were similar to the ages reported in other studies for HSIL.^27^ This study demonstrated that there was no difference in age between women with uVIN and those with NNL.

Our study supports the view that vulvar cancer cannot be differentiated from NNL based on clinical evaluation alone.^26^ Vulvar pruritus, pain, and other gross features of vulvar lesions are not cancer-specific symptoms. This similarity of vulvar cancer signs and symptoms with those of NNL has been cited as a possible reason for the delay in the diagnosis of gynaecologic malignancies by clinicians.^28,29,30^ It is therefore imperative that all vulvar lesions are biopsied as soon as possible, especially among women living with HIV.

Although they have been identified as risk factors of vulvar cancer, the smoking of cigarettes, alcohol use, use of oral contraceptives, and early sexual debut were not associated with VSCC or uVIN in our cohort of participants.^31,32^ Also, the finding that 90% of patients with VSCC had been on ART for over a year supports previous reports that ART does not affect the regression of low- or high-grade lesions and has no effect on the prevention or elimination of HPV infection.^33,34,35,36^

In our study, the mean CD4 count among women with VSCC was above the AIDS diagnosis threshold: 200 cells/µL.^37^ In contrast, in a 2014 study in Botswana, 35% of women living with HIV with vulvar cancer had CD4 counts below 200 cells/µL (range: 68–854).^22^ In another study in Ethiopia in 2018, the range of CD4 counts was above the AIDS diagnosis threshold (230 cells/µL – 600 cells/µL).^27^ Our study was unique in that it showed that there was no association between CD4 count and the diagnosis of VSCC.

This study supports previous findings that itching is the most frequent symptom of uVIN and VSCC^7,38^ and demonstrated that VSCC presents with multifocal growth patterns.^39,40,41^ The finding of concurrent HSIL and a high Ki-67 proliferation index with a diffuse pattern among VSCC was indicative of a bad prognosis.^42^ However, an evaluation of the correlation between patients’ clinical outcomes and these prognostic factors was beyond the scope of this study.

Although polymerase chain reaction showed that HRHPV was present in the biopsy tissue, it did not prove that HRHPV was incorporated into the host genome. Nevertheless, the PCR findings were supported by the histologic examination which detected uVIN, as well as warty and basaloid VSCC, all of which are known to be associated with HRHPV.^5^

The high prevalence of HRHPV in VSCC and HSIL in our cohort of women living with HIV is premised on the extensively described interactions between HPV infection and HIV.^43,44,45,46^ On one hand, women who are infected with HPV have a higher risk of infection with HIV.^36^ On the other hand, HIV-positive persons are at higher risk of developing HPV infection through various mechanisms. One of these mechanisms involves the secretion of a viral transactivator protein and gp120 by HIV-infected cells, which disrupt epithelial tight junctions and enhance intercellular penetration of the HPV to the target cells.^36^ Because of defective immunity in the host, HPV evades the host immunity, and the suppressed immunity further enhances increased HPV viral loads, infection with multiple HPV types, reactivation of latent HPV infection, and persistence of HPV-associated disease.

The prevalence of HRHPV in VSCC in our study (88.9%) was higher than the prevalence reported in Botswana (82.9%).^19^ Worldwide, the prevalence of HPV in VSCC varied from 15.0% to 79.0%.^5^ The prevalence of HPV16 in VSCC in this study was low (44.4%)^47^ compared to that reported by a study in Botswana (82.9%) but similar to the prevalence reported worldwide (29.3% – 50.0%).^22,48,49,50^ We reported a prevalence of 44.4% for the group P3 genotypes (HPV31, 33, 35, 52 and 58) among patients with VSCC. This was lower than the prevalence rate of HPV31 (51.4%) and higher than the prevalence rate of HPV33 (11.4%) reported in a Botswanan study; it was also higher than the prevalence rates reported worldwide (0.8% – 1.7% for HPV31 and 3.3% – 3.5% for HPV33).^48,49^ A further evaluation of the exact genotypes that are prevalent in Zambian patients is imperative. The prevalence of HPV18/45 (5.6%) among patients with VSCC in our study is difficult to compare with other regional studies as such studies measured the prevalence rates of HPV18 and HPV45 separately. For example, the Botswanan study in 2014 reported a 37.1% HPV18 prevalence and an 8.6% HPV45 prevalence.^22^

In this study, the prevalence of HRHPV was high (100%) among HSIL cases and low among LSIL cases (25.0%); the overall prevalence of HRHPV among uVIN cases was 82.4%. This was in keeping with previous studies worldwide that showed that HPV prevalence varied from 52.0% to 100.0% in uVIN.^5^ The 64.7% prevalence of HPV16 among uVIN cases in this study is lower than the 74.6% prevalence reported worldwide among uVIN cases.^5,51^ The presence of HPV group P3 and the absence of HPV18/45 among HSIL cases suggests that HPV31, 33, 35, 52 and 58 may have a higher pathogenic role in causing HSIL than HPV18/45.

Vaccination against HPV is effective in preventing HPV-related cancer. For instance, in Scotland, there were statistically significant reductions in all grades of cervical intraepithelial neoplasia. The vaccine was estimated to be ≥ 80% effective in preventing cervical intraepithelial lesions when administered routinely to girls aged 12–13 years in the targeted population.^52^ Different types of HPV vaccines are available, including the quadrivalent HPV vaccine, Gardasil^®^ 9 (Sanofi Pasteur MSD)/Silgard^®^ (Merck Sharp & Dohme), and the bivalent HPV vaccine, Cervarix^®^ (GlaxoSmithKline Biologicals). In Europe, these vaccines were indicated for the prevention of cervical, vulvar and vaginal premalignant lesions and cervical cancer related to HPV16/18. The quadrivalent HPV vaccine was also indicated against premalignant anal lesions and anal cancer and protected against low-risk HPV6/11, which are responsible for about 90% of genital warts.^53^ Zambia initially introduced the HPV vaccine on a pilot basis in 2013 and by 2020, the country had integrated it into the routine immunisation programme using a system that offers two doses of the quadrivalent HPV vaccine.^54^ Based on the preliminary findings of our study, more research needs to be conducted to determine the specific genotypes of HRHPV that are prevalent in the population affected by either cervical or vulvar cancer to inform current vaccination efforts.

### Limitations

Even though University Teaching Hospital is Zambia’s highest level referral centre for the treatment of major ailments, a multicentre study would have represented the country’s population better. The responses given by the participants may have been negatively influenced by their recall bias. One downside with our methodology is that GeneXpert reports pooled HPV genotypes and hence we could not report the specific genotypes in the P3, P4 or P5 groups. Furthermore, we did not evaluate viral load, which is considered a better marker of immunologic status. Also, we did not successfully determine viral DNA incorporation into the host genome using either p16 immunohistochemistry or in situ hybridisation.

### Conclusion

VSCC was associated with women aged above 40 years. The prevalence of HRHPV in VSCC and HSIL was high. We recommend that women living with HIV, especially women aged above 40 years, should have a close clinical follow-up with routine evaluation of the vulva and biopsy of all lesions. We recommend a multicentre study to further evaluate vulvar neoplastic lesions in Zambia and determine other genotypes other than HPV16 to inform current vaccination efforts. Human papillomavirus vaccination of adolescent girls should be supported as a public health approach to the elimination of vulvar neoplasia.
